# From the Cochrane Library: Foam Surfaces for Preventing Pressure Ulcers

**DOI:** 10.2196/34112

**Published:** 2023-01-05

**Authors:** Yvonne Nong, Torunn Sivesind, Robert P Dellavalle

**Affiliations:** 1 College of Human Medicine, Michigan State University Flint, MI United States; 2 School of Medicine, University of Colorado Aurora, CO United States

**Keywords:** pressure ulcer, decubitus ulcer, foam surface, prevention, systematic review, wound, dermatology, ulcer, skin, pressure sore, bedsore, bed sore

As an essential component of the total body skin exam, dermatologists should recognize early signs of pressure ulcer development [1] and provide evidence-based preventive measures for patients who are at high risk. Pressure (decubitus) ulcers are common injuries to the skin and underlying soft tissue, resulting from prolonged pressure or shear force. Severe pressure ulcers may deepen, causing localized damage to muscles, tendons, and bones. Patients with limited mobility, systemic comorbidities, or decreased skin integrity [2] are most susceptible. Commonly affected areas include the lower back, sacrum, hips, and heels. A 2021 Cochrane review [3] offers a comprehensive review of the evidence regarding foam as a support surface.

The review [3] included all publications prior to the literature search (November 2019). Included were 29 studies encompassing over 9500 people considered at risk for, or who currently have, pressure ulcers that compared foam mattresses with surfaces like gel, air cells, or water bags. Participants were mainly from acute care settings; the median study sample size was 101 participants, with an age range of 47.0-85.3 years. Support surfaces were categorized into either reactive or alternating pressure types. The primary outcome was pressure ulcer incidence, and secondary outcomes (patient comfort, adverse events, health-related quality of life, and cost-effectiveness) were also evaluated. The relative risk (RR) of pressure ulcer development with foam surfaces compared to alternating pressure air surfaces was 1.59 (95% CI 0.86-2.95); despite failing to reach statistical significance, the authors reported this finding as low-certainty evidence that foam surfaces may increase the risk of pressure ulcer development compared to alternating pressure air surfaces. Many surface comparisons demonstrated very low–certainty evidence [3].

In evaluating time-to-pressure ulcer development, one study suggested that viscoelastic foam surfaces with densities of 40-60 kg/m^3^ may decrease new pressure ulcer development over 11.5 days compared to lower-density foam surfaces of 33 kg/m^3^. Another study assessed solid versus convoluted foam surfaces and found that the latter may decrease the risk of pressure ulcer development over 1 month. Despite these conclusions, both studies had low-certainty evidence. Furthermore, the authors reported low confidence in the effect estimate of secondary outcome measures overall. One such measure was cost-effectiveness, for which one study provided moderate-certainty evidence suggesting that alternating pressure air surfaces may be superior to foam surfaces in preventing pressure ulcers when such factors are considered [3].

This review is among four that examine specific surface types for pressure ulcer prevention. Further research is needed given the low strength of evidence regarding various surface types in preventing decubitus ulcers. Factors to consider in future studies are an emphasis on time-to-event outcomes, adverse effects, and the cost-effectiveness of various surface types. Notably, more than half (58.6%) of the studies analyzed were considered to have a high risk of bias, mostly concerning the nonblinding of participants, personnel, and outcome assessments; therefore, careful attention to reducing the risk of bias should also be an element of future studies. Trials should be designed to minimize the risk of detection bias, for example, by using digital photography and by blinding adjudicators of the photographs to group allocation.
